# Inertial sensors for smartphones navigation

**DOI:** 10.1186/s40064-015-1572-8

**Published:** 2015-12-30

**Authors:** P. Dabove, G. Ghinamo, A. M. Lingua

**Affiliations:** Politecnico di Torino, Corso Duca degli Abruzzi 24, 10129, Turin, Italy; Telecom Italia, Turin, Italy

**Keywords:** Smartphones, MEMS, Inertial sensors, Image based navigation, Indoor positioning

## Abstract

The advent of smartphones and tablets, means that we can constantly get information on our current geographical location. These devices include not only GPS/GNSS chipsets but also mass-market inertial platforms that can be used to plan activities, share locations on social networks, and also to perform positioning in indoor and outdoor scenarios. This paper shows the performance of smartphones and their inertial sensors in terms of gaining information about the user’s current geographical locatio n considering an indoor navigation scenario. Tests were carried out to determine the accuracy and precision obtainable with internal and external sensors. In terms of the attitude and drift estimation with an updating interval equal to 1 s, 2D accuracies of about 15 cm were obtained with the images. Residual benefits were also obtained, however, for large intervals, e.g. 2 and 5 s, where the accuracies decreased to 50 cm and 2.2 m, respectively.

## Background

Image recognition based (IRB) positioning is a good technology for navigation in Global Navigation Satellite System (GNSS) denied environments such as indoors (Krishnamurthy 2015), or urban canyon conditions where GNSS accuracy is poor (tens of meters accuracy; Dabove and Manzino 2014; Lee et al. 2014). To be able to exploit IRB positioning not only the battery optimization is crucial (Faragher and Harle 2015; Fister et al. 2013), which involves the minimization of the frame rate, but also the size of the database with the environment images. In fact due to the large memory size, this database cannot be easily stored on mobile devices. To counteract the database issue, a cloud architecture can be exploited where the device acquires the image used for the localization procedure and the cloud performs all the necessary operations to estimate the parameters of the camera (Woodman and Harle 2008; Yuan et al. 2014). In this architecture it is thus necessary to compensate for the latencies of calculation by the cloud (De Agostino et al. 2010). Latencies, however, can be also present in terminal based SW implementations and should also be compensated for.

To overcome the reduction of the frame rate and latencies compensation, inertial (INS) platforms built with MEMS (Micro Electro-Mechanical Systems) technology can be used and considered (Afzal et al. 2011; Aicardi et al. 2014; Niu et al. 2015).

Two different approaches are possible for the inertial navigation with MEMS technology (Tian et al. 2015; Kunze et al. 2009): the pedestrian dead reckoning (PDR; Yunye et al. 2011) or the INS integration approach which integrates equations regarding inertial measurements for the positions and attitude estimation (Frank et al. 2014; Hatami and Pahlavan 2005; Michel et al. 2015). In this integration approach, inner variables, such as accelerometer biases, gyro drift and 3D inertial velocity, are estimated by combining INS information with an absolute external source that provides absolute reference information (Kalliola 2011; Schatzberg et al. 2014). Usually the absolute external source is represented by GNSS instruments that provide periodic absolute position information. In this work we investigated how GNSS instruments can be replaced by IRB positioning. IRB provides absolute attitude information together with the position directly as the input of the equation system. Consequently it cannot be considered as an inner state of the equation system as in the usual Kalman filter approach (Deng et al. 2015; Li et al. 2011; Kraus 1997).

The PDR technique simply considers the estimation of a step length (or walking speed) (Lee et al. 2015) and a course over ground (or direction of walking) (Cliff Randell and Muller 2003; Ladetto et al. 2001). Hybridizing the PDR with the IRB technique, the step length model and the actual length of the trajectory can be obtained (Yang and Huang 2015). This, thus, makes the PDR system more precise and increases the intervals between two subsequent localization images, which have about 30 cm of uncertainty (Lingua et al. 2014; Piras et al. 2014).

User experience of step detection algorithms shows that occasionally the initial step is not detected. If we suppose that the mean foot length is 25 cm, the step length is about 0.7 m and the accuracy of correction using PDR is 3 % of the walked distance, it is not possible to detect errors less than one step, which is equal to 1 m. It should also be underlined that displacements due to hand movements or other events different from regular steps can not be detected. PDR can only be used for hand-held sensors and not for wheeled vehicles with mounted cameras (Tung and Shin 2015).

**Fig. 1:**
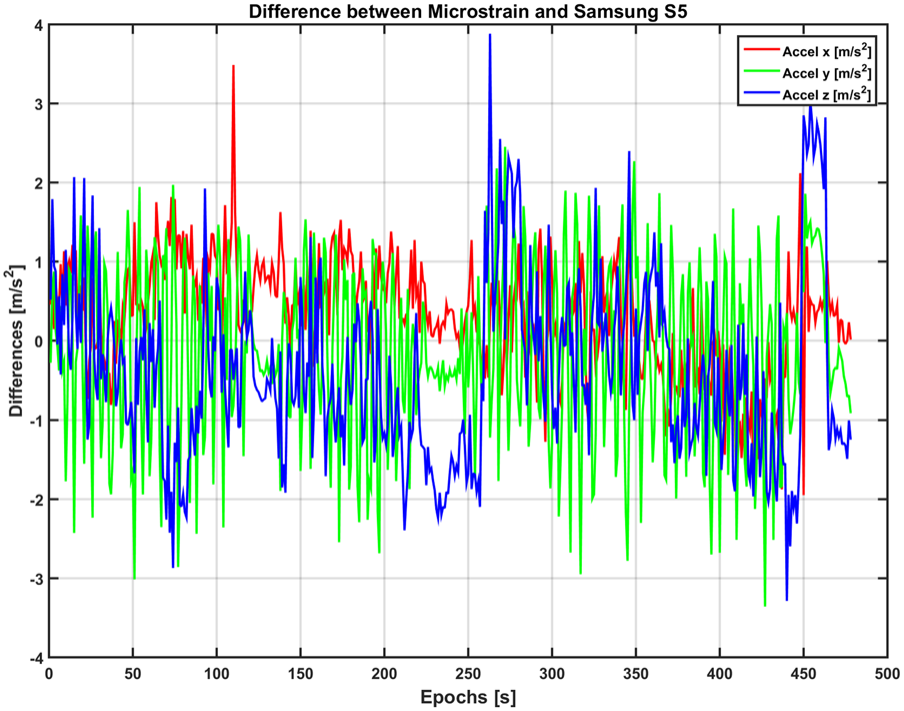
Differences of accelerations and angular velocities between Microstrain and Samsung S5

In this study we investigated the positioning using only inertial MEMS instruments. Thus, the goal of this work is to analyze the precisions and accuracies obtainable with these instruments for positioning and indoor navigation purposes when MEMS technology is used together with IRB positioning. This is achieved by fusing IRB position and attitude measurement with INS measurements in terms of three axial acceleration and three axial angular velocity (Fig. 1). The Microstrain platform is considered as a representative instrument of this category. Accelerations and angular velocity measurements are integrated to provide real-time relative position and relative attitude information, while inner INS variables (velocity at the starting point, accelerometer biases and gyro drift) are estimated using absolute IRB positioning inputs (position and attitude). The IRB positioning periodically provides absolute reference values regarding positions and attitudes, with a latency that needs to be adjusted. We calculate this time interval in order to evaluate how often a picture needs to be taken in order to stay within a defined error bound. For pedestrian navigation, this error bound is usually assumed to be equal to 2 m. More stringent requirements of accuracy are shown for particular applications such as augmented reality, in particular in indoor environments (Masiero et al. 2014) with limited dimensions, such as applications in commercial sales support (smart retail).

We analyse the characteristics (in terms of accuracy, noise, and reliability) of the sensors installed inside the smartphones considered and the benefits of wavelet filtering techniques, such as data obtained by the inertial internal phones. The tests results are also described in terms of accuracy achieved for different time intervals (1, 2 and 5 s) between two different IRB positioning procedures.

## Analysis of inertial sensors installed on some high-level smartphones

The IRB navigation procedure can also be adopted for mass-market devices; the most popular sensors available today are smartphones that now include many useful sensors for geomatics applications.

The performance of the most well-known devices available was tested in Piras et al. 2014. Various internal sensors have been embedded, such as digital cameras and GNSS receivers (Jones et al. 2015), inertial platforms based on gyroscopes, accelerometers (Hekler et al. 2015) and magnetometers and RFID systems for smartphone devices (Jain and Kanhangad 2015).

In this paper, only the Samsung Galaxy S5 and iPhone 4 were considered and their technical information is shown in Table 1. This choice is made considering the devices available today in our geomatics lab.

**Table 1:** Devices and their principal characteristics

		
Name	Samsung Galaxy S5	iPhone 4
Cost (€)	500	400
OS	Android 4.4.2 TouchWiz UI KitKat	iOS 7.0.6
CPU	Adreno 330	Apple4—800 MHz
Digital camera Resolution (Mpx)	16	5
Type of lens	CMOS	CMOS
A-GPS	Yes	Yes
GNSS receiver	Broadcom	Broadcom—BCM4750
Inertial platform	Yes	Yes

These devices include sensors whose characteristics are needed to obtain a good and feasible positioning. In particular, to achieve a positioning with a photogrammetric approach, it is fundamental to characterize the noise level of the inertial sensors and to calibrate the camera, thus, removing the lens distortion.

### Data acquired through smartphones and wavelet filtering

The main aspect that needs to be considered when a new sensor is analysed is the stability of the internal inertial sensors, whose performance is not usually declared by the manufacturers. We, thus, performed a 6-h static test to acquire the raw data (angular velocity and accelerations) of each smartphone to analyse the stability.

**Fig. 2:**
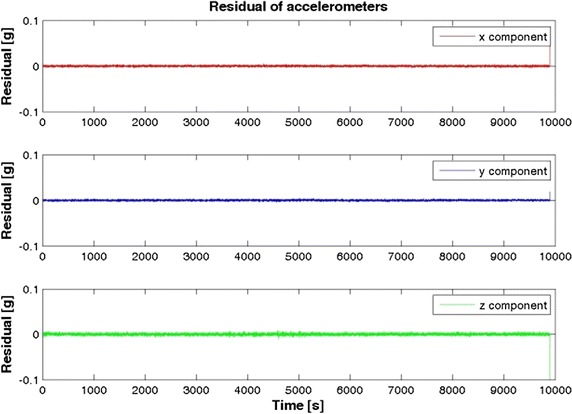
Acceleration residuals for Samsung Galaxy S5

**Fig. 3:**
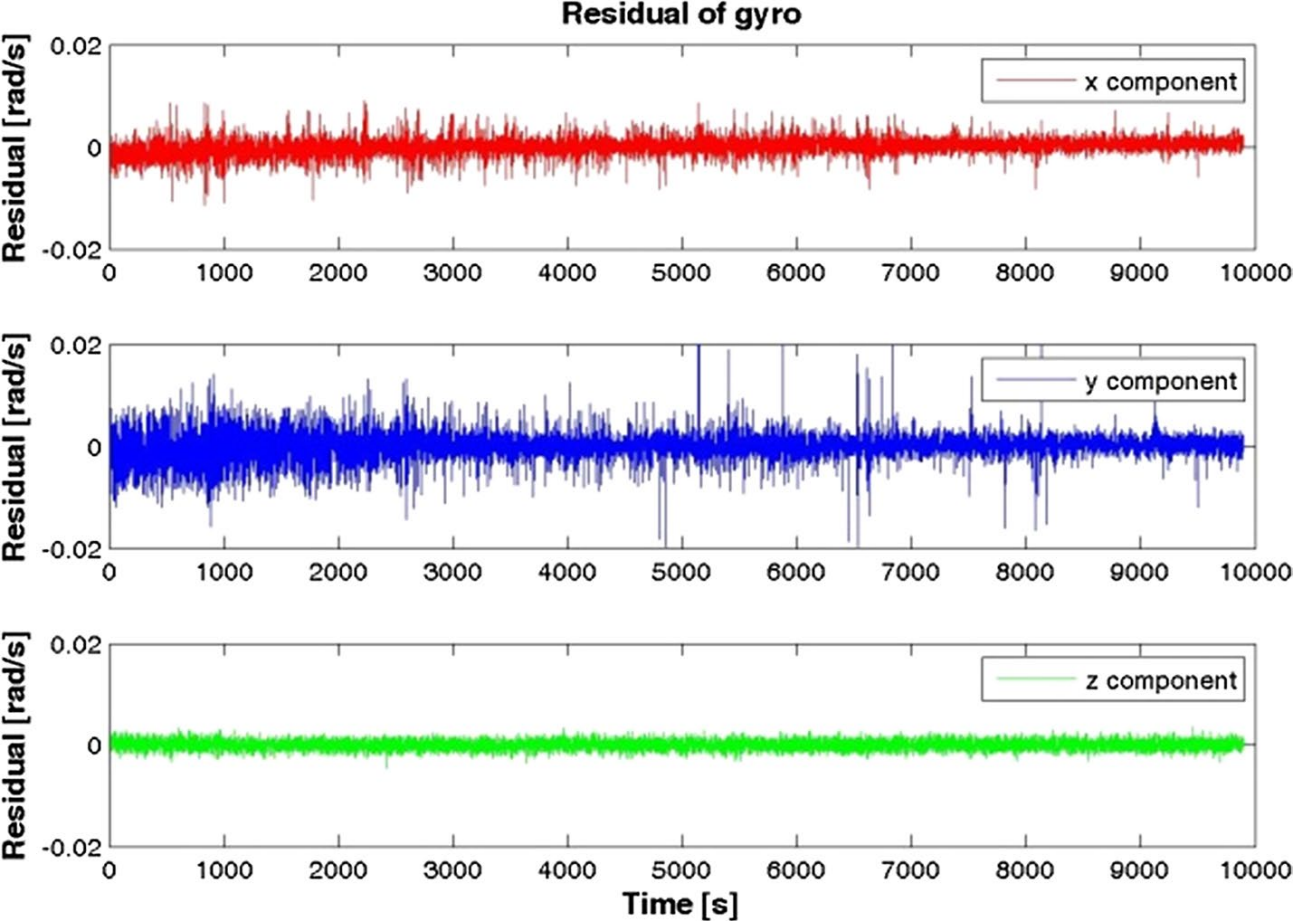
Gyroscope residuals for Samsung Galaxy S5

**Fig. 4:**
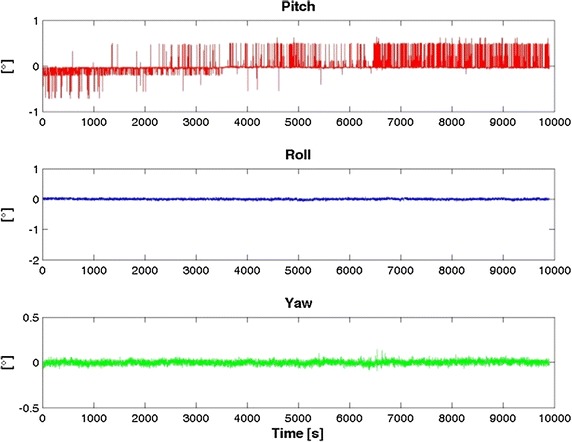
Roll, Pitch and Yaw stability for Samsung S5

Figures 2, 3 and 4 show the result of the Samsung S5 inertial platform analysis.

**Fig. 5:**
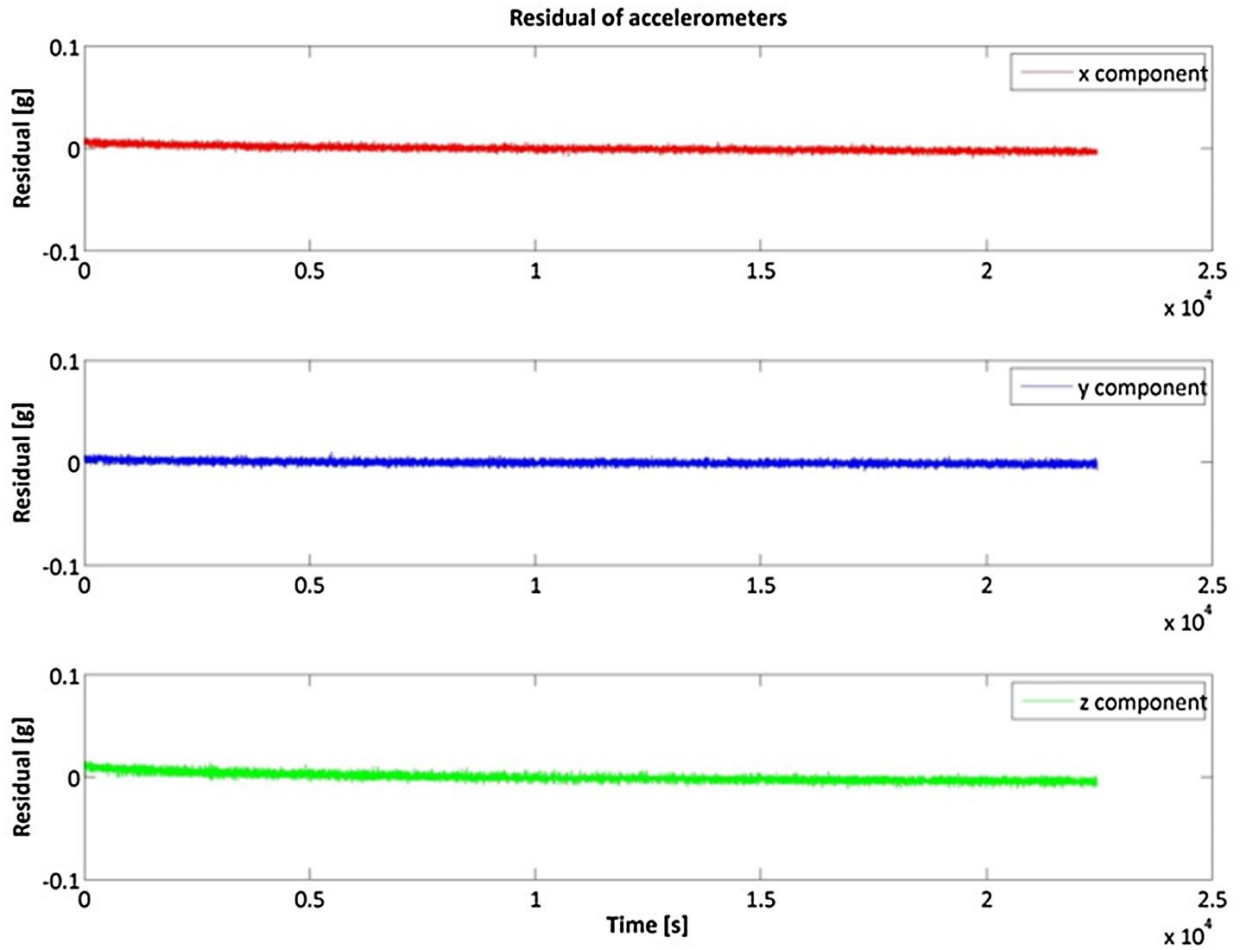
Acceleration residuals for iPhone 4

**Fig. 6:**
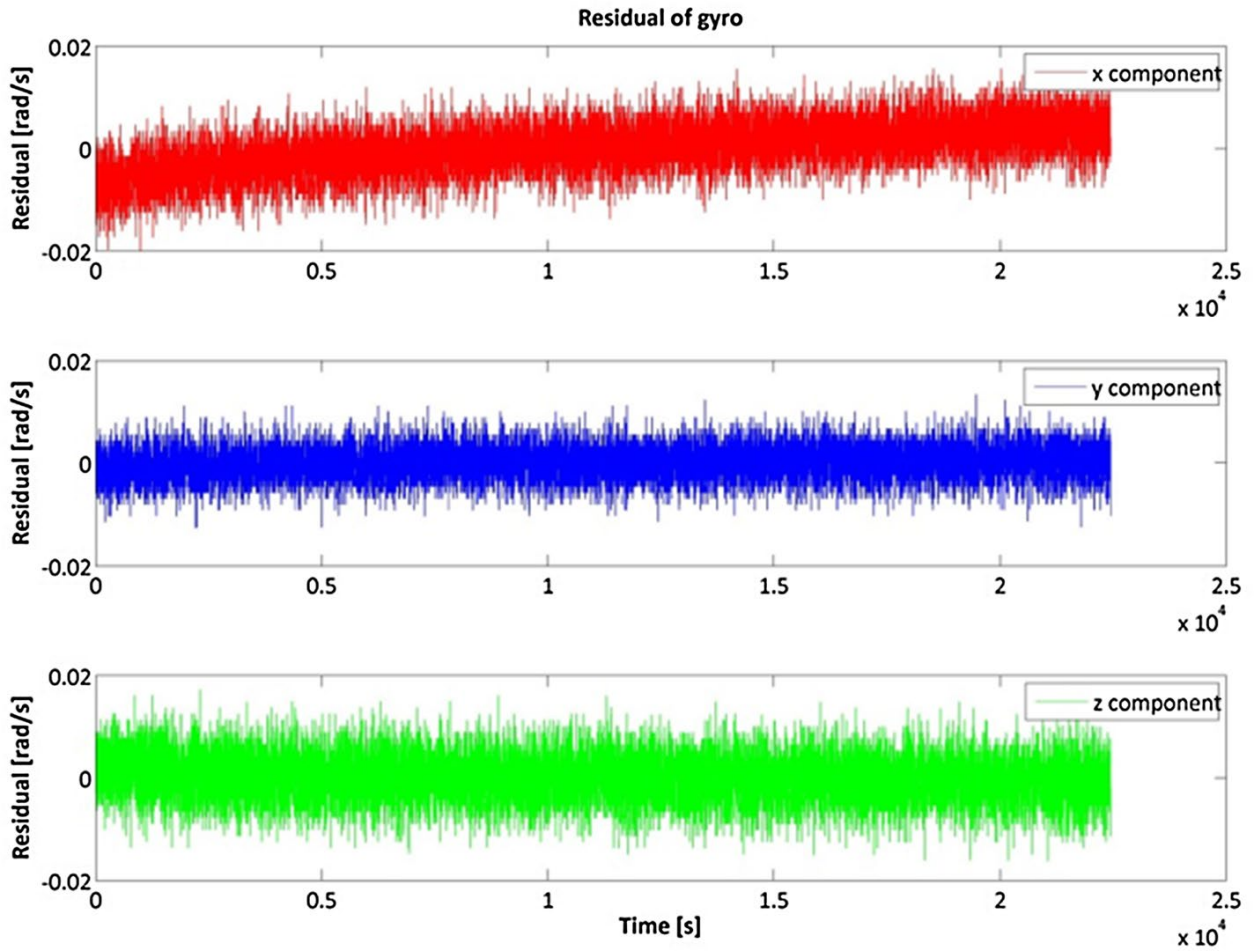
Gyroscope residuals for iPhone 4

**Fig. 7:**
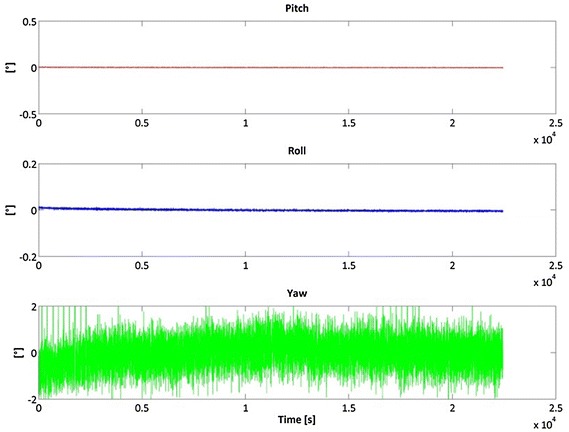
Roll, Pitch and Yaw stability for iPhone 4

The stability and the performance of the iPhone 4 are described in Figs. 5, 6 and 7.

Table 2 highlights that in general the iPhone 4 accelerometers are slightly better than the Samsung S5, while it is the opposite for the gyro components. Note, however, that our goal was not to determine which smartphone is the “best” but which is more useful for IRB purposes.

**Table 2:** RMS of the inertial sensors

RMS	X	Y	Z
Samsung S5			
Acceleration (g)	0.0046	0.0061	0.0002
Gyro. (rad/s)	0.0017	0.0028	0.0009
iPhone 4			
Acceleration (g)	0.0028	0.0024	0.0042
Gyro. (rad/s)	0.0048	0.0032	0.0043

The inertial measurements acquired by these types of sensors are very noisy, due to the MEMS technology of the sensors themselves (Werner et al. 2014; Shin et al. 2014). Thus, for a “good” positioning, this noise needs to be removed before using the raw data. For the inertial sensors, the noise of the data can be corrected through filtering (Rousseeuw and Leroy 1987). Filtering entails using the wavelets, which are signal representations of the oscillations of a waveform. There are different kinds of these representations, but in our case, the Daubechies wavelets were chosen to correct the data and, in particular, we used a Daubechies 4 at a level 7, as previously described in Piras et al. (2014).

**Fig. 8:**
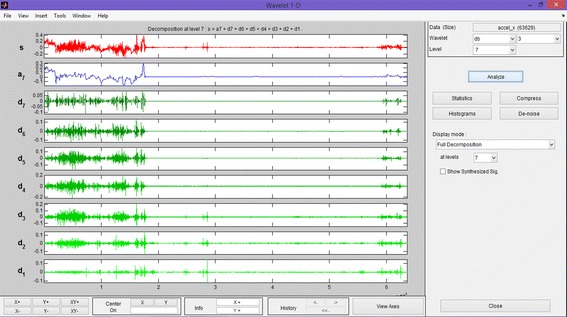
Wavelet denoising

**Fig. 9:**
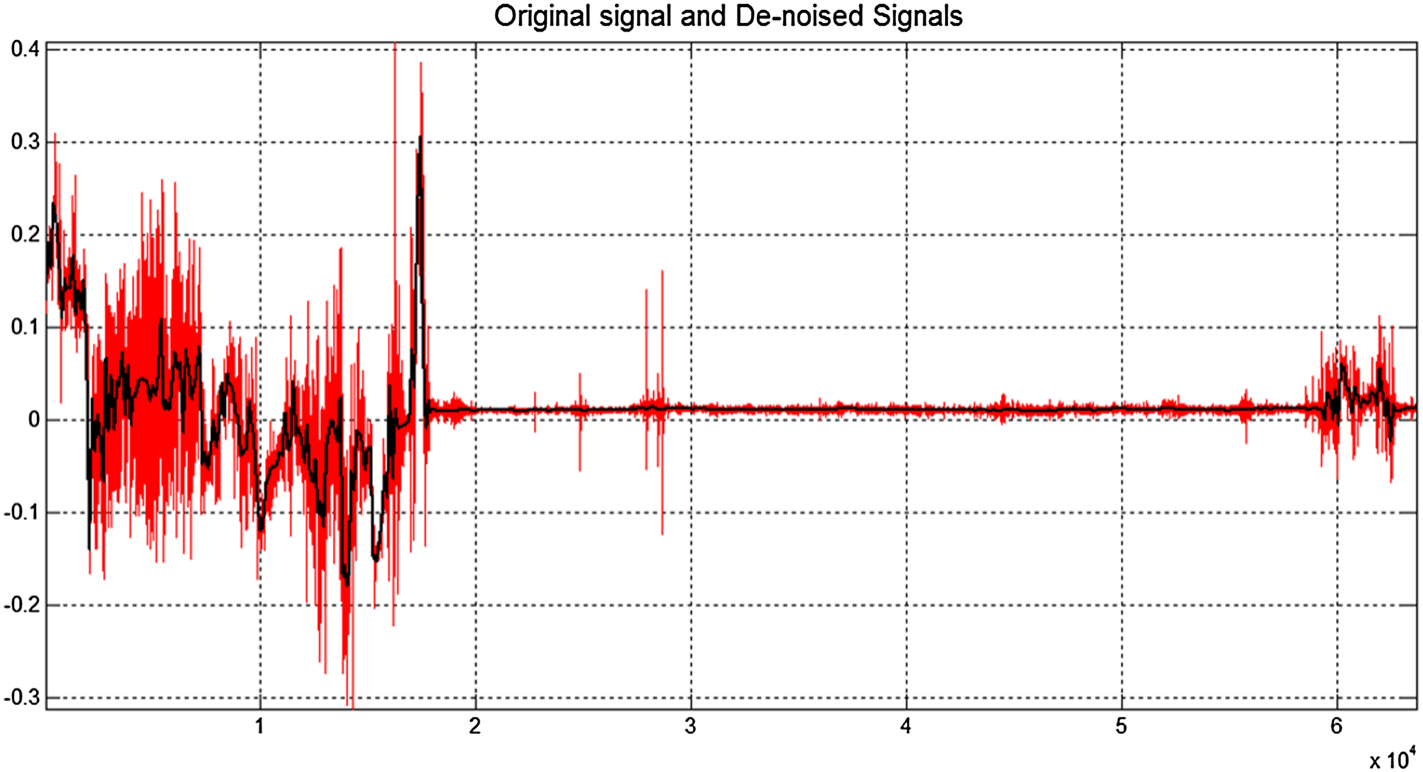
Original (*red*) and denoised (*black*) signals

Figure 8 shows the wavelet denoising approach (Rousseeuw and Leroy 1987) considering the Matlab toolbox (but a similar procedure is implemented by us and the results obtained are the same). Figure 9 shows an example of the signal after the wavelet filtering: original signal is marked in red while the denoised signal is shown in black).

## The reference sensor

A smartphone is useful for many activities (in addition to mere communication). Although accurate positioning with this device has been extensively investigated; some key information on the navigational purposes with INS instruments is not known, such as the reference systems of the internal sensors.

Various experiments were, thus, performed to determine the reference systems of the internal inertial platforms of smartphones, by performing calibration tests using the external reference inertial measurement unit (IMU).

**Fig. 10:**
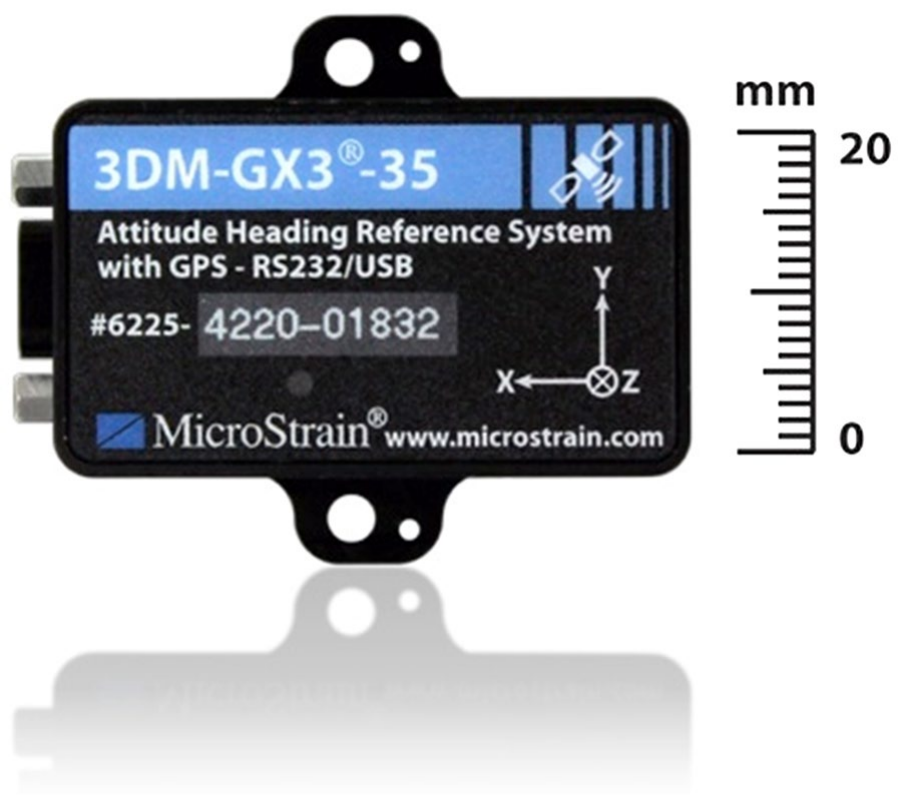
External IMU platform with a known internal reference system

On a non-magnetic plate, the two smartphones shown in Table 1 and the external inertial sensor, whose internal reference system is known and imprinted on the top part of the same, as shown in Fig. 10, were installed. This sensor is the Microstrain 3DM-GX3-35TM, whose technical characteristics and performance in terms of stability and accuracies are shown in Fig. 10.

**Fig. 11:**
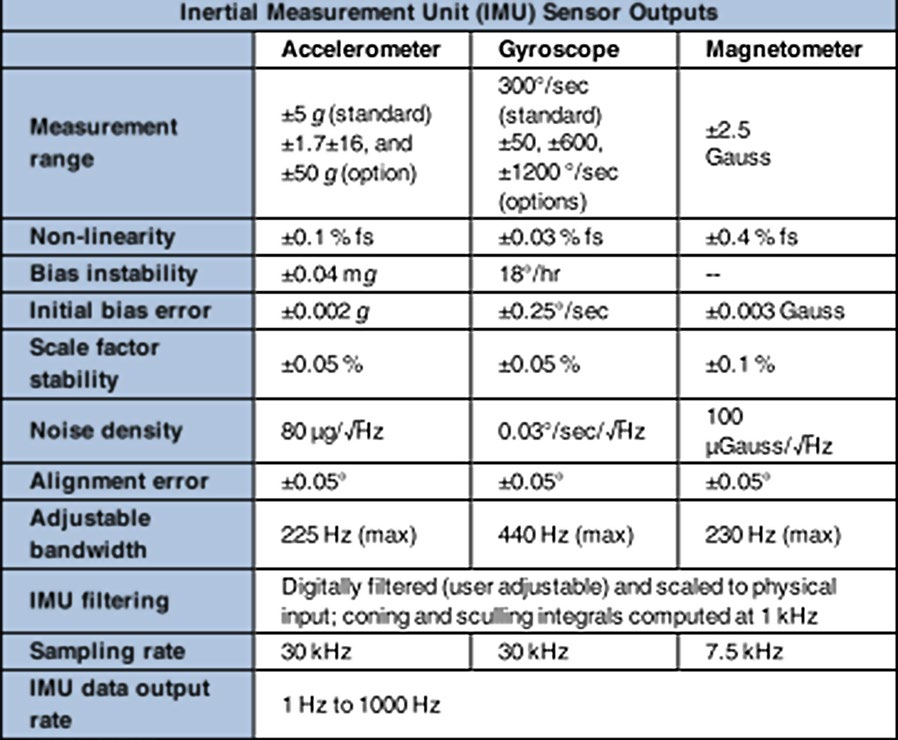
External IMU platform datasheet (source: http://files.microstrain.com/3DM-GX3-35_Datasheet_%288400-0034%29.pdf)

By performing rotations of known angles and displacements, the determination of the internal reference systems of each smartphone was possible, which is essential for the surveying and data processing phases (Fig. 11).

**Fig. 12:**
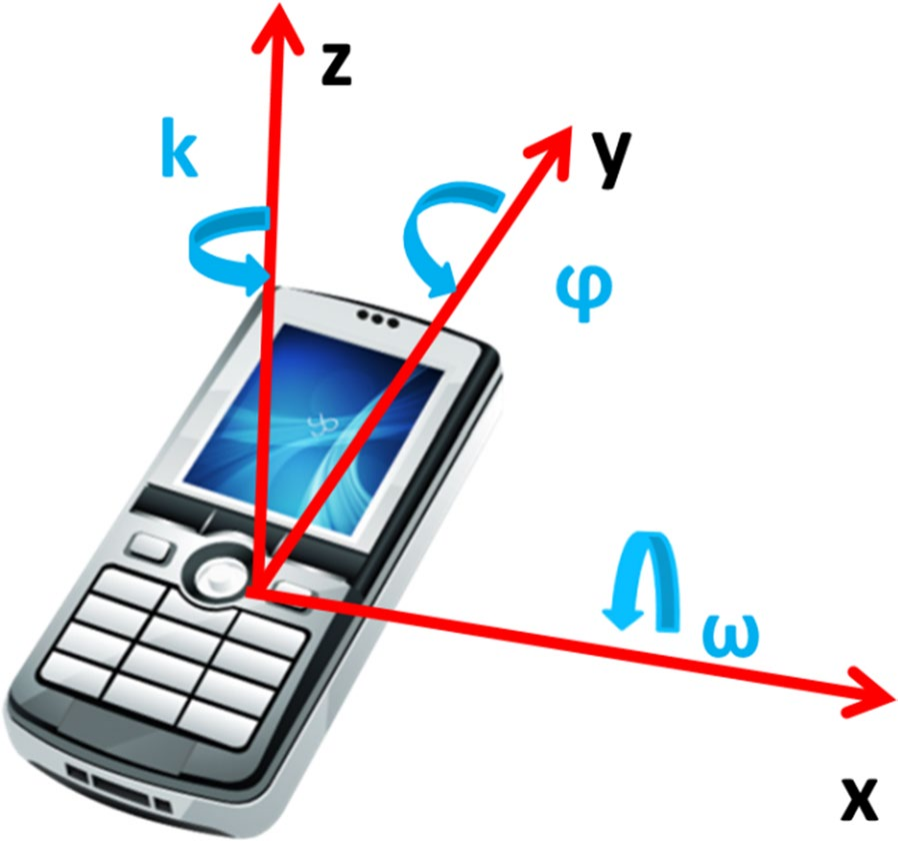
Rotation angles and attitude estimation

The second step of the work was based on writing software that performs real-time positioning through images based localization and inertial sensors installed within the smartphone (Fig. 12).

To analyze the quality of the positioning achieved with the internal sensors, a software was written in Matlab, considering a system with six states (three position components of position and three attitude angles); three different time windows were considered for the dynamic estimation of the inner-state system, due to biases, drift and speed of motion, which are useful parameters for predicting the position and attitude at the generic epoch *n* with INS instrumentation, together with the positions and attitudes obtained at epoch *n* − 1.

## The methodology for relative position and attitude estimation based on INS measurements

In the scenario of hybrid IRB and INS positioning, the states are periodically estimated with IRB positioning which provides absolute values for both attitude and position (Woodman 2007). INS measurements, which provide relative variations in position and attitude, are used in the period between two images, in order to have a continuous positioning for navigation purposes. When a new image is taken, new values of absolute position and attitude can be obtained directly from IRB, so it is possible to estimate drift and biases of the INS instrument, which can be used for the following epochs.

In this context considering IRB + INS techniques, real-time position and attitude are estimated by evaluating the following equations:

1 $$x_{k} = x_{n} + \mathop \sum \limits_{k = 1}^{t} \left( {\left( {v_{n} + \mathop \sum \limits_{h = 1}^{k} \left( {R_{h} \times \left( {a_{h} - bias_{n} } \right) \times d{\text{t}}} \right)} \right) \times d{\text{t}}} \right) + \frac{1}{2}\mathop \sum \limits_{k = 1}^{t} \left( {R_{k} \times \left( {a_{k} - bias_{n} } \right) \times d{\text{t}}^{2} } \right),$$

2 $$\varphi_{k} = \varphi_{n} + \mathop \sum \limits_{k = 1}^{t} \left( {R_{k} \times \left( {\omega_{k} - drift_{n} } \right) \times {\text{d}}t} \right),$$

where x_k_, vector of coordinates; v_k_, the vector of velocities; R_h_, rotation matrix from body to navigation frame; a_k_, the acceleration vector; dt, time interval; bias, bias vector; drift, drift vector; $\varphi$_k_, attitude expressed as three orientation angles; ω_k_, angular velocity vector; k, index for the sequence of positions based on sensor measurement rate; n, sequence of INS epochs between two IRB epochs (n is a periodic subset of k values).

It is therefore necessary to estimate the bias used in (1) and the drift used in (2) at each step.

If the position is evaluated in three consecutive epochs *n*, *n* + 1 and *n* + 2 with IRB fix (i.e. epochs with positions and attitudes estimated by the IRB technique) and the bias is assumed to be constant over the two time intervals, the three components of velocity at time *n* (v_n_) and the bias of the three accelerometers (one for each axis) can be estimated by solving the following linear system of six linear equations:

$$\left\{ {\begin{array}{*{20}c} {X_{n + 1} - X_{n} = V_{n} \times {\text{d}}t + \mathop \sum \limits_{k = 1}^{T} \left( {\mathop \sum \limits_{h = 1}^{k} \left( {R_{h} \times \left( {a_{h} - bias_{n} } \right) \times {\text{d}}t} \right)} \right) \times {\text{d}}t} \\ {X_{n + 2} - X_{n} = V_{n} \times 2{\text{d}}t + \mathop \sum \limits_{k = 1}^{2T} \left( {\mathop \sum \limits_{h = 1}^{k} \left( {R_{h} \times \left( {a_{h} - bias_{n} } \right) \times {\text{d}}t} \right)} \right) \times 2{\text{d}}t} \\ \end{array} } \right.,$$

where X represents the position vector.

For the drift estimation, attitude measurements coming from two consecutive epochs *n* and *n* + 1 are needed: a linear system in three unknowns (one for each gyro sensor mounted on a three axial device) needs to be solved:

$$\varphi_{n + 1} - \varphi_{n} = \mathop \sum \limits_{k = 1}^{t} \left( {R_{k} \times \left( {\omega_{k} - drift_{n} } \right)} \right) \times {\text{d}}t,$$

where $\varphi$ represents the attitude expressed as three orientation angles.

After an initial alignment phase of about 3 s, during which the initial attitude of the smartphone is estimated, some IMU data (accelerations and angular velocities) are acquired, ensuring that they are all turned in the navigational reference system.

IMU data are also acquired from the Microstrain sensor integrating a GPS receiver, in terms of absolute position and orientation, together with IMU three axis acceleration and three axis angular velocities. An initialization phase of 3 s is required by the IMU and GPS data fusion algorithm for absolute attitude estimation.

On the basis of Woodman (2007) and Beauregard and Haas (2006), a strapdown system was implemented so that the orientation and the speed of the device are calculated and combined to compute the direction of motion.

Note that in this phase of the prototyping algorithm, measures from image positioning are replaced with ones from the output data available with the Microstrain sensor (position and attitude).

**Fig. 13:**
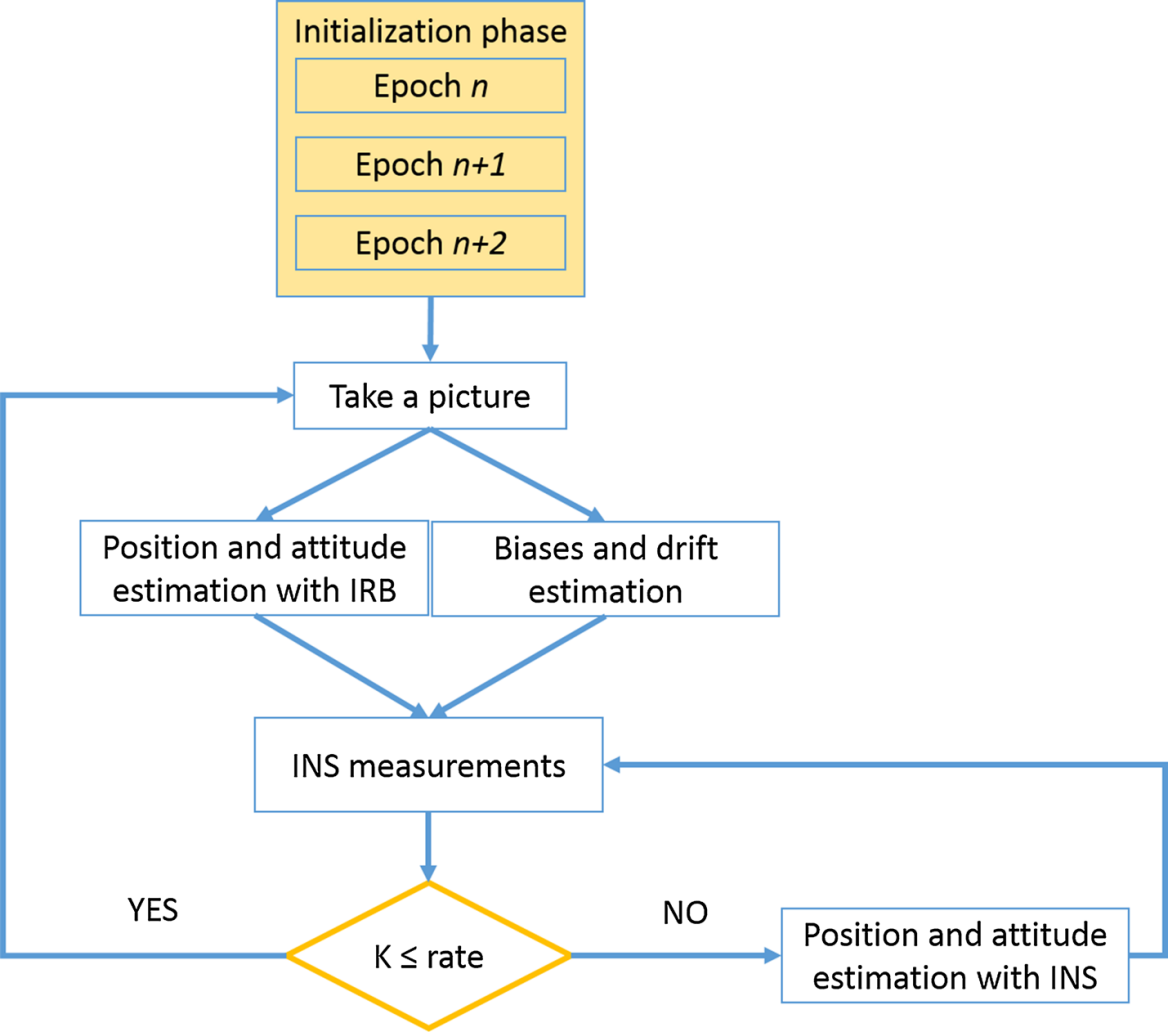
The procedure flowchart

If there are measures arising from the total station (or later from images), the software can use this information source to estimate acceleration and attitude biases along the three components and determine the new rotation matrix that can be used at the next epoch. When the total station position information is exploited, attitude information is not available and Microstrain information is used. Figure 13 shows a flowchart of the procedure.

A non-optimal simplified approach with respect to the Kalman filter process was followed. This is because the characterization parameters of the inertial platform, the stability of the biases and drift estimation are not required by our system.

The objective of the study, however, was to evaluate how INS technology can be applied to compensate for the latency due to image positioning using the cloud database, as described in (Lingua et al. 2014). Thus, the ground truth and the attitude (obtained automatically by the Kalman filter as output) for the inertial instrument was replaced by simulating external data of the image localization procedure (attitude and velocity obtained by the IRB), whose accuracy is described in Lingua et al. (2014).

## Test setup

**Fig. 14:**
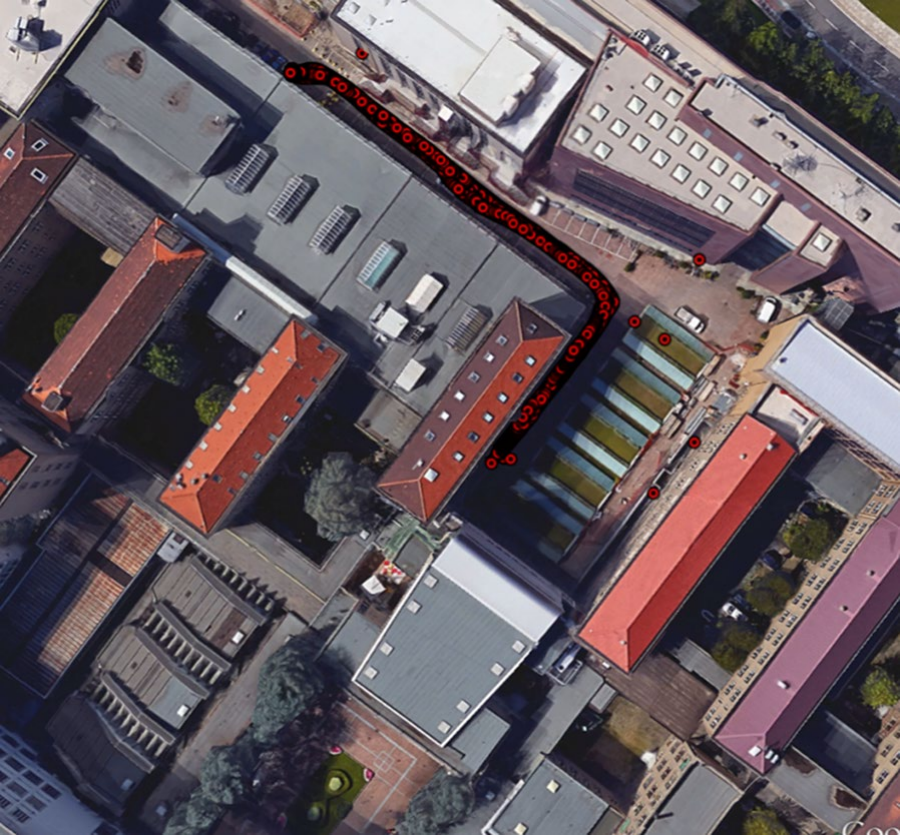
Test site and track

The tests were set up in a courtyard in our campus (Fig. 14). The track (red line in Fig. 14) was performed especially in an area with many windows and high repeatability of the modules.

**Fig. 15:**
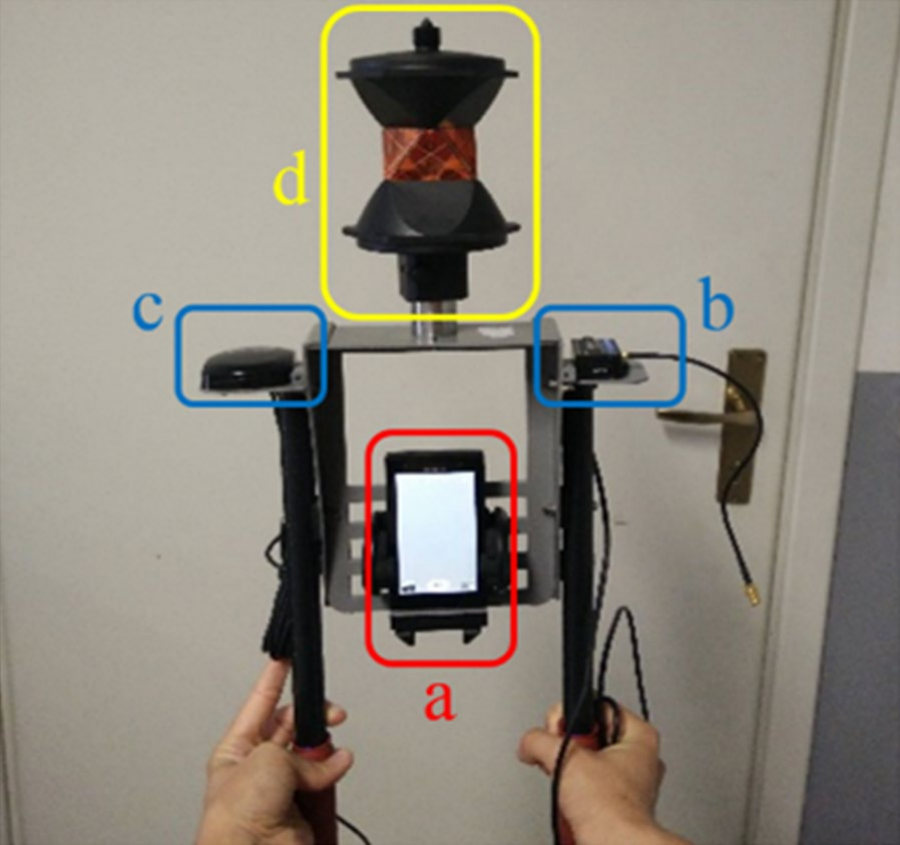
The system used to acquire the data

The test was performed by walking on the same path using the two different smartphones (a) mounted on a special support (Fig. 15), designed by our Geomatics Laboratory, which supports:

- An inertial platform IMU-MEMS Microstrain 3DMGX35 (b) with external antenna (c);
- A 360° retroflector (d).

During the tests each smartphone sensor recorded its own inertial sensor data using a dedicated app: for the smartphone with the android (Tarapata 2015) operating system (Samsung S5) the “AndroSensor” app was used, which provides graphical information and text (.csv) output, while for the iPhone, the “SensorLog” app was used.

The reference trajectory was defined by tracking the position of the smartphone continuously with a total station, through the retroflector. Thus, the position of the support was measured with millimetre accuracy.

In addition, the data from the external IMU (Microstrain) was stored using a computer, to have reference values for the attitude.

For this application different types of data were acquired from the devices, in particular:

- Samsung S5: images;
- iPhone 4: images and videos.

No substantial differences in precision and accuracy can be obtained considering images or videos, thus in this paper only results obtained with images are shown.

After the tests, all the IMU data files concerning each sensor, the images of the tracks and the reference data for the comparison between the estimated and the real solution were processed.

## Results

The primarily goal of these tests was to assess the accuracy of the estimated position by varying the rate in which the displacement was evaluated based on the data of the inertial platform.

The Microstrain inertial platform was chosen as an inertial sensor because it is the type of inertial sensor that will be installed on board the next generation of smartphones, considering both the costs of production and size. The effect of denoising with wavelets was also evaluated: the use of wavelet filtering did not add any benefits because the raw measures were too noisy and a shape variation of the bias was not identified.

**Fig. 16:**
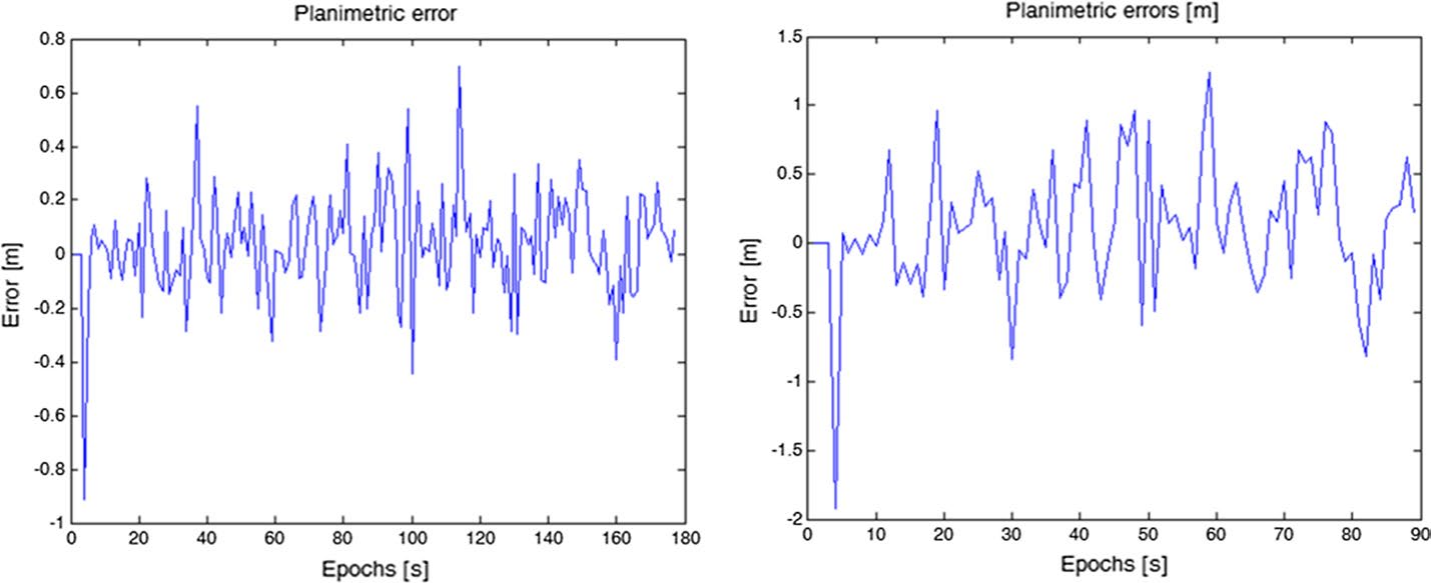
Planimetric errors considering intervals equal to 1 s (*left*) and 2 s (*right*) between two successively known positions estimated by total station

Regarding the navigation, the results can be considered as satisfactory: considering an interval of 1 s (Table 3) between images, the mean planimetric error was 21.3 cm at 67 % of reliability, while at 95 % this error was 37 cm (Fig. 16).

**Table 3:** Results obtained without bias estimation but with wavelet denoising

	1 s			2 s			5 s		
	Mean	67 %	95 %	Mean	67 %	95 %	Mean	67 %	95 %
E	0.144	0.163	0.359	0.390	0.480	0.960	1.655	2.262	4.252
N	0.131	0.140	0.400	0.369	0.389	1.148	1.533	1.711	3.864

**Table 4:** Results obtained with drift estimation coming from images

	1 s			2 s			5 s		
	Mean	67 %	95 %	Mean	67 %	95 %	Mean	67 %	95 %
N	0.14	0.16	0.38	0.42	0.47	1.27	2.60	2.70	5.80
E	0.14	0.16	0.44	0.42	0.50	1.78	2.60	2.90	6.50

When the positioning obtained with an interval of 2 s between the images is analyzed, the mean planimetric error increases to 61 cm at 67 % and 1.49 m at 95 %.

The tables show that in these tests the measurements of angular velocities were already adjusted for drift, according to the estimation made by the Microstrain itself, and it was not necessary to estimate the drift of the gyro at this stage, because the attitude measurements are already correct.

**Fig. 17:**
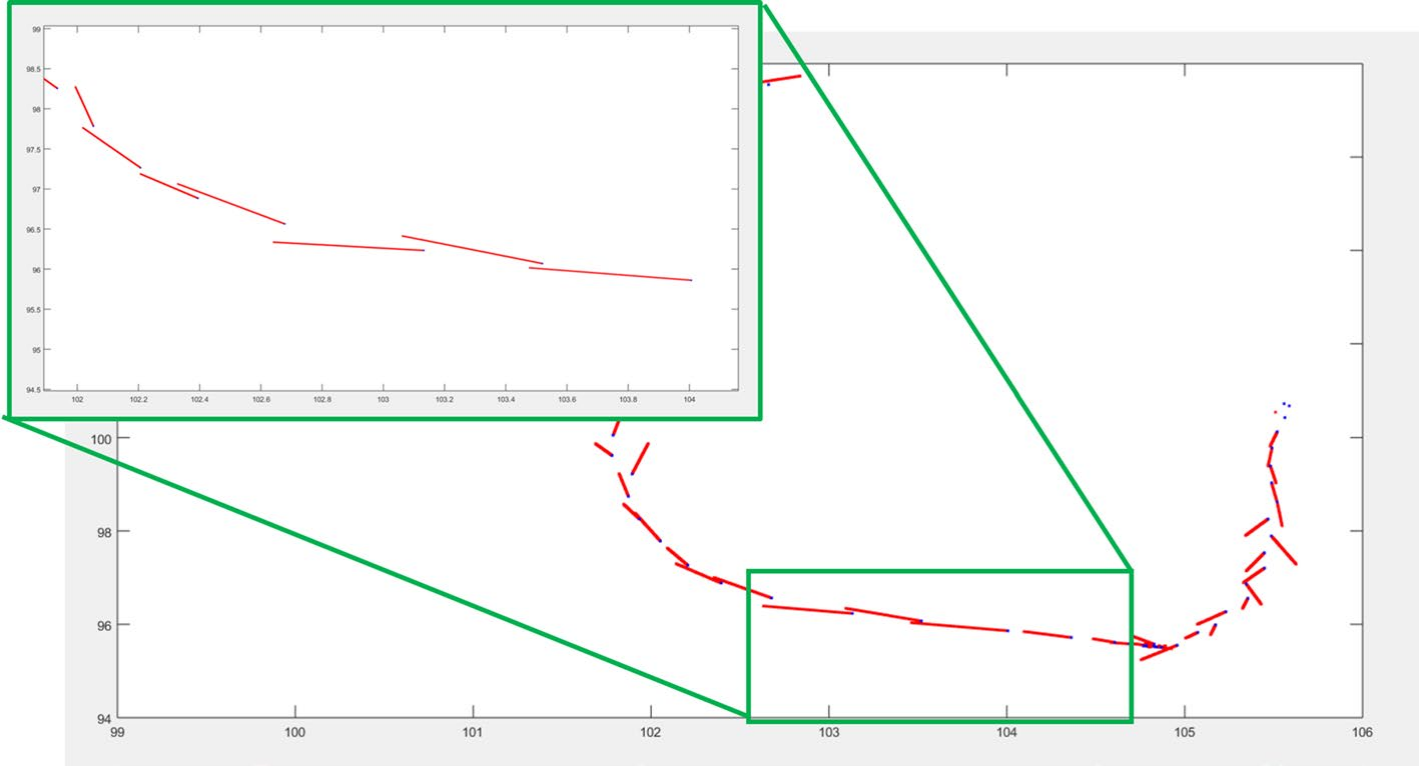
Positioning obtained with Microstrain sensor and an interval of 2 s between total station positions

The results obtained with an interval between two epochs (equal to 2 s) with total station are shown in Fig. 17.

While in the previous cases the results were obtained considering the attitude and the drift estimation provided by the Microstrain sensor, the accuracy results were obtained with different drift estimation modes. Table 4 shows the procedure described in “Test setup” which can be considered as an optimal hybridization strategy for IRB and INS. If 1 s is considered, there was a mean planimetric error of 14 cm and an error of about 20 cm assuming a 67 % confidence level and 60 cm with a confidence level of up to 95 %. This is an acceptable result for pedestrian navigation. If the 2 s interval is considered, the mean planimetric error is 0.5 and 1.5 m with a confidence level of 67 and 95 %, respectively. It is still acceptable although it does not match the requirements of augmented reality for indoor environments. For intervals of 5 s the error increases up to 4 m with a 67 % confidence level which is not acceptable either for navigation application or augmented reality.

**Table 5:** Results obtained with drift estimation obtained by GPS

	1 s			2 s			5 s		
	Mean	67 %	95 %	Mean	67 %	95 %	Mean	67 %	95 %
N	0.13	0.14	0.38	0.30	0.28	1.03	1.5	1.84	4.33
E	0.11	0.12	0.37	0.31	0.33	1.08	1.7	1.36	4.39

Tables 4 and 5 show no substantial differences from Table 6 in relation to intervals of 1 s.

**Table 6:** Results obtained without bias estimation and wavelet denoising

	1 s			2 s			5 s		
	Mean	67 %	95 %	Mean	67 %	95 %	Mean	67 %	95 %
E	0.130	0.148	0.353	0.387	0.485	0.960	1.673	2.221	4.158
N	0.130	0.141	0.412	0.380	0.409	1.162	1.574	1.677	3.952

In Table 7 the drift estimated by the Microstrain is considered under a hypothesis of a position estimation available every second. The results at 1 s are comparable to the one with pure image simulated information. Improvements for larger intervals are due to a better gyro drift estimation executed by the Microstrain every 1 s.

**Table 7:** Results obtained without drift estimation

	1 s			2 s			5 s		
	Mean	67 %	95 %	Mean	67 %	95 %	Mean	67 %	95 %
N	0.23	0.23	0.56	0.8	1.02	1.99	6.11	5.53	13.86
E	0.23	0.23	0.56	0.86	0.99	2.19	5.4	6.1	13.87

These tables highlight that the results obtained with images are nearly the same as those obtained with the bias and drift estimation from the Microstrain sensor. In this context, 1 or 2 s is the best intervals for positioning estimation with smartphones.

This approach, based on the decoupling of the estimated accelerometer biases from the estimated drift of the gyros, using measures of attitude not available in the usual techniques when GPS and INS are coupled, provides comparable results with those obtained by the Kalman filter implemented on the Microstrain, under the condition that the update rate is the same (i.e. 1 s). This is highlighted by comparing Table 6 (bias, drift and velocity estimated by Kalman filter) and Table 5 (inner state estimation with decoupled approach).

However, to use a Kalman filter it is necessary to make assumptions about the dynamics of motion and have knowledge of the noise of accelerometers and gyros, as well as the drift variation of the INS, none of which are required in the described approach.

## Conclusions

Our tests demonstrated that INS measurements can be coupled with image based positioning to reduce latencies of a cloud architecture, if these latencies are less than 2 s. Under this hypothesis, the 1.5 m error boundary is always respected, although an error of this order may lead to various drawbacks for augmented reality. In fact many applications in indoor environments are characterized by limited spaces where the camera position should be more precise in order to provide more accurate information on the localization. For navigation applications, a latency interval of 2 s can be compensated by INS instruments with an error less than 1.5 m.

The PDR is a more efficient technique for a longer frame rate. On the other hand, INS with the approach described in this work may be more useful in a short time range, because after 1 s the obtained 2D accuracy is 14 cm while after 5 s it becomes 1.5 m, if the attitude estimated by the Microstrain is considered.

Considering the attitude and drift estimation obtained with images, the results obtained are very similar. The estimated drift is effective if the updating intervals are in the order of 1 s. Residual benefits are obtained, however, also for larger intervals (e.g. 2, 5 s). With this study we have demonstrated that the automatic techniques coupled with an INS instrument is useful for indoor navigation because errors are less than 1 m with intervals of about 2 s between two images, even if it could be not optimized for the worst cases (e.g. some indoor environments where there are few details that brings to have less descriptors). An alternative solution can be represented by the range camera coupled with INS instruments, that will be investigated in the future.
